# Kikuchi-Fujimoto Disease in Human Leukocyte Antigen Partially Matched Siblings: A Case Study of Familial Susceptibility

**DOI:** 10.7759/cureus.51010

**Published:** 2023-12-23

**Authors:** Atsushi Isoda, Kenichi Tahara, Munenori Ide

**Affiliations:** 1 Department of Hematology, Iryohojin Hoshiiin, Maebashi, JPN; 2 Department of Hematology, Maebashi Red Cross Hospital, Maebashi, JPN; 3 Department of Pathology, Maebashi Red Cross Hospital, Maebashi, JPN

**Keywords:** familial susceptibility, human leukocyte antigen, sibling, histiocytic necrotizing lymphadenitis, kikuchi-fujimoto disease

## Abstract

Kikuchi-Fujimoto disease (KFD) is a rare and self-limiting disorder that predominantly affects young individuals of Asian descent. This case report describes familial KFD in partially human leukocyte antigen (HLA)-matched siblings. An adolescent male presented with cervical lymphadenopathy and elevated lactate dehydrogenase (LDH) levels, diagnosed by biopsy as KFD; approximately one year later, his sister presented with similar symptoms. Both siblings were found to carry the HLA-DPB1*0202 allele, which is commonly associated with KFD. These cases highlight a genetic component in KFD and encourage further genetic research to delineate the pathogenesis of the disease.

## Introduction

Kikuchi-Fujimoto disease (KFD), also known as histiocytic necrotizing lymphadenitis, is a rare and self-limiting condition found predominantly in young individuals of Asian descent. Patients with KFD typically present with a febrile illness lasting several days to weeks, accompanied by generalized malaise and the presence of tender or swollen cervical lymph nodes [1,2]. The definitive diagnosis of KFD is usually established by lymph node biopsy [3], although a watchful waiting approach may also be considered given the potential for spontaneous disease remission within one to four months. While the exact cause of KFD remains elusive, the notable ethnographic bias in its incidence may indicate an increased T-cell-mediated immune response to various non-specific stimuli in a genetically susceptible population [4]. Here we present cases of siblings who sequentially developed KFD approximately one year apart and describe their human leukocyte antigen (HLA) typing analysis.

## Case presentation

Case 1 (brother)

**Figure 1:**
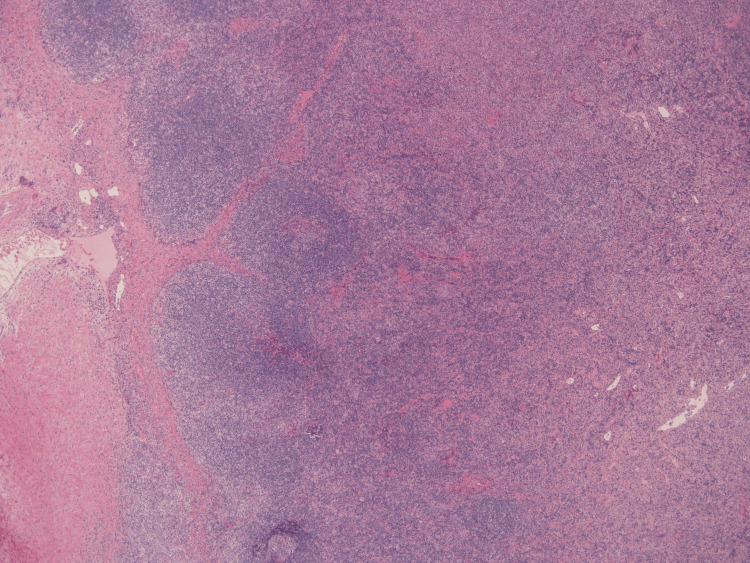
Biopsy specimens of the right cervical lymph nodes in case 1 (hematoxylin and eosin staining, ×40 magnification) Paracortical geographic eosinophilic areas with preservation of lymphoid follicles.

**Figure 2:**
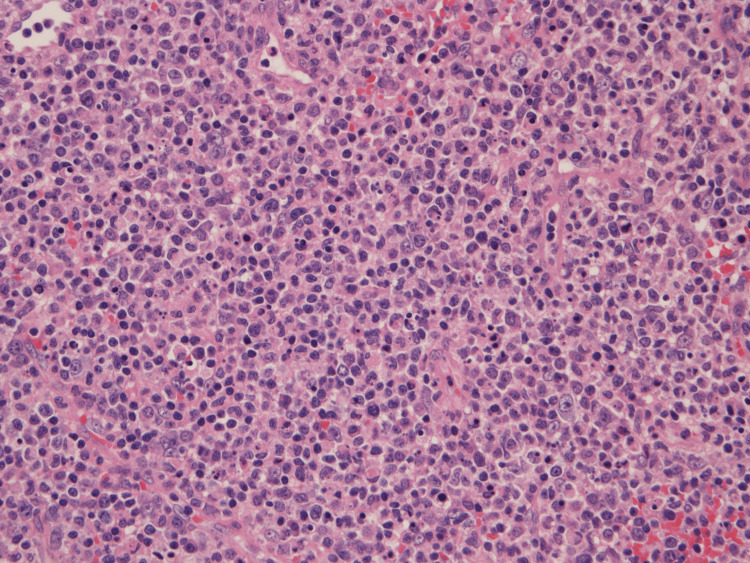
Biopsy specimens of the right cervical lymph nodes in case 1 (hematoxylin and eosin staining, ×400 magnification) Abundant nuclear debris and proliferation of reactive lymphocytes and phagocytic histiocytes. Large lymphocytes are also observed sporadically, but these findings are not neoplastic.

A 16-year-old Japanese male presented to our clinic with a two-week history of right cervical lymphadenopathy. He had been in excellent health since birth. He reported fatigue but denied fever, respiratory symptoms, or joint pain. On physical examination, multiple lymph nodes, each measuring 25 mm, were palpable on the right side of his neck. There was no enlargement of the tonsils or extra-neck lymph nodes, and there was no hepatosplenomegaly. Laboratory tests revealed an elevated lactate dehydrogenase (LDH) level, while the complete blood count was within normal limits. Infectious and autoimmune tests were negative (Table 1). Two weeks after the initial presentation, a right cervical lymph node biopsy was performed. Biopsy specimens revealed paracortical geographic necrosis with abundant karyorrhectic nuclear debris. There is a proliferation of reactive lymphocytes and phagocytic histiocytes, indicating KFD's immunologic response (Figures 1, 2). Although mildly elevated transaminases persisted for two months, the patient's symptoms, other than lymphadenopathy, remained minimal. Consequently, conservative management was chosen, and steroid therapy was not administered. Lymphadenopathy completely resolved after five months.

**Table 1:** Laboratory parameters of siblings WBC, white blood cell; RBC, red blood cell; TP, total protein; T-Bil, total bilirubin; AST, aspartate aminotransferase; ALT, alanine aminotransferase; LDH, lactate dehydrogenase; ALP, alkaline phosphatase; BUN, blood urea nitrogen; CRE, creatinine; CRP, C-reactive protein; CH50, 50% hemolytic complement; T-SPOT, T-cell spot of tuberculosis assay; SIL-2R, soluble IL-2 receptor; CMV, cytomegalovirus; EBV, Epstein-Barr virus; VCA, virus capsid antigen; EBNA, Epstein-Barr nuclear antigen.

	Case 1 (Brother)	Case 2 (Sister)	Reference range
WBC (/μL)	4080	2330	3900–9800
Neutrophil (%)	57	37	30–78
Lymphocyte (%)	34	53	18–60
Monocyte (%)	7	5	3–10
Eosinophil (%)	2	5	0–5
Basophil (%)	0	0	0–1.2
RBC (×10^6^/μL)	5.15	4.49	4.27–5.20
Hemoglobin (g/dL)	14.6	12.9	13.5–17.6
Hematocrit (%)	42.0	39.3	39.8–51.8
Platelets (×10^4^/μL)	24.9	20	13.1–36.2
TP (g/dL)	7.4	8.0	6.7–8.3
T-Bil (mg/dL)	0.4	0.4	0.3–1.2
AST (U/L)	40	30	13–33
ALT (U/L)	36	22	8–42
LDH (U/L)	389	469	119–229
ALP (U/L)	115	78	115–359
BUN (mg/dL)	12.6	11.2	8–22
CRE (mg/dL)	0.89	0.61	0.6–1.1
Na (mEq/L)	139	141	138–146
K (mEq/L)	4.3	4.2	3.6–4.9
Cl (mEq/L)	102	105	99–109
CRP (mg/dL)	0.43	0.56	0-0.14
Anti-nuclear antibody	1:40	<1:40	<1:40
Anti-DNA antibody (IU/mL)	<2.0	<2.0	<6.0
Anti-SS-A (Ro) antibody (U/mL)	<1.0	<1.0	<10.0
CH50 (CH50/mL)	58.8	65.0	25-48
T-SPOT	negative	negative	negative
SIL-2R (U/mL)	677	520	122-496
IgG (mg/dL)	1149	1233	861-1747
IgA (mg/dL)	245	235	93-393
IgM (mg/dL)	71	80	33-183
Ferritin (ng/mL)	266	153	male: 30-310, female: 3-120
CMV-IgG	79.3	195.0	<6.0
CMV-IgM	0.25	0.17	<0.85
EBV-VCA-IgG	40	40	<1:40
EBV-VCA-IgM	<10	<10	<1:40
EBNA	40	40	<1:40
Bartonella-IgG	negative	negative	negative
Bartonella-IgM	negative	negative	negative
Toxoplasma-IgG	negative	negative	negative
Toxoplasma-IgM	negative	negative	negative

Case 2 (sister)

**Figure 3:**
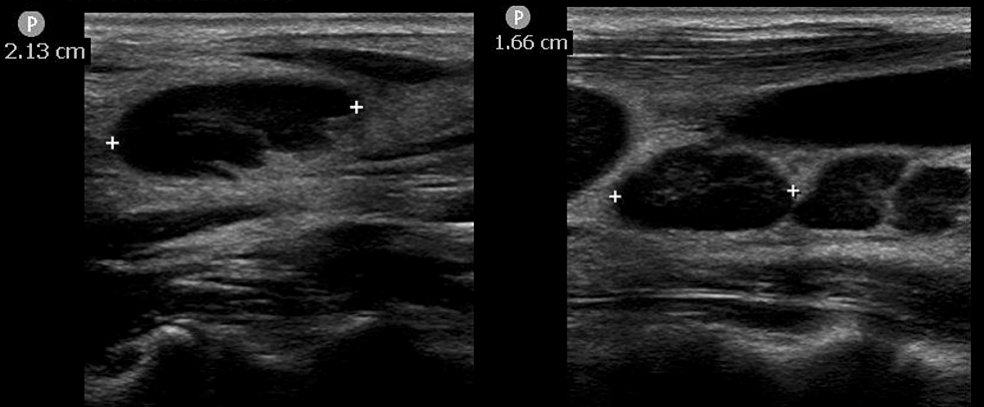
Ultrasonographic imaging of the right cervical lymph nodes in case 2 Multiple lymph nodes less than 25 mm with preserved hilum, suggesting reactive lymphadenopathy.

Eleven months after the onset of case 1, an 18-year-old female who was the sister of case 1 presented to our clinic with a one-week history of right cervical lymphadenopathy. She had been healthy since birth. She reported fatigue, occasional low-grade fevers at night, and headaches but denied any respiratory symptoms or joint pain. On physical examination, multiple lymph nodes approximately 20 mm in size were palpable on the right side of her neck. Ultrasonographic imaging revealed lymph nodes with preserved pulmonary hilum and increased vascularity, consistent with reactive lymphadenopathy (Figure 3). Similar to her brother, there was no enlargement of the tonsils or other lymph nodes, and hepatosplenomegaly was absent. Laboratory tests revealed an elevated LDH level and a decreased white blood cell count. As in her brother's case, infectious disease and autoimmune tests were negative (Table 1). Because of concerns about scarring, a biopsy was not performed, and the patient was closely monitored without treatment. Mildly elevated transaminases persisted for one month, and the lymphadenopathy resolved after four months.

HLA typing of siblings

HLA typing was performed on the siblings at a later date, as shown in Table 2. It is noteworthy that the siblings showed complete concordance in HLA class I (HLA-A, B, C) and partial concordance in HLA class II (HLA-DR, DQ, DP). Furthermore, both individuals carried the HLA-DPB1*0202 allele, which has been reported to be a common HLA type associated with KFD [5].

**Table 2:** HLA typing of siblings Siblings showed complete HLA class I (HLA-A, B, C) and partial HLA class II (HLA-DR, DQ, DP) concordance.

	Case 1 (Brother)	Case 2 (Sister)
HLA-A	02:07:01, 24:02:01	02:07:01, 24:02:01
HLA-B	40:02:01, 46:01:01	40:02:01, 46:01:01
HLA-C	01:02:01, 03:04:01	01:02:01, 03:04:01
HLA-DRB1	04:10:03, 08:03:02	08:02:01, 08:03:02
HLA-DRB3/4/5	4*01:03:01	
HLA-DQA1	01:03:01, 03:03:01	01:03:01, 03:01:01
HLA-DQB1	04:02:01, 06:01:01	03:02:01, 06:01:01
HLA-DPA1	01:03:01. 02:02:02	02:02:02
HLA-DPB1	02:02:01, 03:01:01	02:02:01, 05:01:01

## Discussion

The exact cause of KFD is still unknown. Despite the implication of various infectious agents such as viruses [6-8], bacteria [9], and parasites [10], a definitive causative agent for KFD remains unidentified. However, the increased prevalence of KFD in individuals of Asian descent suggests that genetic factors, such as HLA, may play an important role. In particular, HLA class II genes, the DPA1*01 and DPB1*0202 alleles, have been reported to be significantly more common in individuals affected by KFD [5].

In the present report, we documented cases of siblings who successively developed KFD approximately one year apart. These siblings had complete HLA matching at class I loci and partial matching at class II loci, along with a common HLA-DPB1*0202 allele. Further suggesting a genetic association, their mother experienced similar self-resolving lymphadenitis in adolescence, although her HLA typing was not performed. Sibling cases of KFD have occasionally been reported, some with complete or partial HLA matching [11-13]. However, no common HLA type has been found in these case reports, probably due to the limited number of cases.

Because of the familial clustering of cases, environmental factors such as exposure to certain pathogens or chemicals cannot be completely ruled out. However, given the significant interval between the onset of disease in the siblings, their lack of pet ownership, and the absence of similar symptoms in other household members, it seems unlikely that infectious or physicochemical factors alone caused the disease. Rather, the sequential occurrence of the disease in siblings with nearly identical genetic constitutions, including HLA markers, suggests the possibility of a genetic predisposition influenced by subtle or as yet unidentified environmental factors.

## Conclusions

This report highlights the importance of familial predisposition in KFD. Future research, including HLA typing and investigation of additional genetic markers, is essential to clarify the specific genetic contributors to KFD and their role in disease susceptibility and development.
